# MK3 controls Polycomb target gene expression via negative feedback on ERK

**DOI:** 10.1186/1756-8935-5-12

**Published:** 2012-08-07

**Authors:** Peggy Prickaerts, Hanneke EC Niessen, Emmanuèle Mouchel-Vielh, Vivian EH Dahlmans, Guus GH van den Akker, Claudia Geijselaers, Michiel E Adriaens, Frank Spaapen, Yoshihiro Takihara, Ulf R Rapp, Frédérique Peronnet, Jan Willem Voncken

**Affiliations:** 1 Department of Molecular Genetics, GROW School for Oncology and Developmental Biology, Maastricht University, Universiteitssingel 50, 6229ER, Maastricht, The Netherlands; 2 BiGCaT Bioinformatics, Maastricht University, Universiteitssingel 50, 6229ER, Maastricht, The Netherlands; 3 Laboratoire de Biologie du Développement UMR 7622, Centre National de la Recherche Scientifique, Université Pierre et Marie Curie-Paris 6, 9 Quai Saint-Bernard, 75005, Paris, France; 4 Department of Stem Cell Biology, Research Institute for Radiation Biology and Medicine, Hiroshima University, 1-2-3, Kasumi, Minami-ku, Hiroshima, Japan; 5 Department of Molecular Biology, Max Planck Institute of Biochemistry, Am Klopferspitz 18, D-82152, Martinsried, Germany

**Keywords:** Polycomb, MAPKAPK3, MK3, ERK, epigenetic, feedback, dynamic

## Abstract

**Background:**

Gene-environment interactions are mediated by epigenetic mechanisms. Polycomb Group proteins constitute part of an epigenetic cellular transcriptional memory system that is subject to dynamic modulation during differentiation. Molecular insight in processes that control dynamic chromatin association and dissociation of Polycomb repressive complexes during and beyond development is limited. We recently showed that MK3 interacts with Polycomb repressive complex 1 (PRC1). The functional relevance of this interaction, however, remained poorly understood. MK3 is activated downstream of mitogen- and stress-activated protein kinases (M/SAPKs), all of which fulfill crucial roles during development. We here use activation of the immediate-early response gene *ATF3*, a *bona fide* PRC1 target gene, as a model to study how MK3 and its effector kinases MAPK/ERK and SAPK/P38 are involved in regulation of PRC1-dependent *ATF3* transcription.

**Results:**

Our current data show that mitogenic signaling through ERK, P38 and MK3 regulates *ATF3* expression by PRC1/chromatin dissociation and epigenetic modulation. Mitogenic stimulation results in transient P38-dependent H3S28 phosphorylation and ERK-driven PRC1/chromatin dissociation at PRC1 targets. H3S28 phosphorylation by itself appears not sufficient to induce PRC1/chromatin dissociation, nor *ATF3* transcription, as inhibition of MEK/ERK signaling blocks BMI1/chromatin dissociation and *ATF3* expression, despite induced H3S28 phosphorylation. In addition, we establish that concomitant loss of local H3K27me3 promoter marking is not required for *ATF3* activation. We identify pERK as a novel signaling-induced binding partner of PRC1, and provide evidence that MK3 controls *ATF3* expression in cultured cells via negative regulatory feedback on M/SAPKs. Dramatically increased ectopic wing vein formation in the absence of *Drosophila* MK in a *Drosophila* ERK gain-of-function wing vein patterning model, supports the existence of MK-mediated negative feedback regulation on pERK.

**Conclusion:**

We here identify and characterize important actors in a PRC1-dependent epigenetic signal/response mechanism, some of which appear to be nonspecific global responses, whereas others provide modular specificity. Our findings provide novel insight into a Polycomb-mediated epigenetic mechanism that dynamically controls gene transcription and support a direct link between PRC1 and cellular responses to changes in the microenvironment.

## Background

Mitogen- and stress-activated protein kinase (M/SAPK) signaling pathways relay environment-to-gene information and enable physiologically appropriate cellular responses [1]. MK3 is an interaction partner of extracellular signal-regulated kinase (ERK) and P38 and is targeted by all three M/SAPK signaling cascades [2]; these phosphorylation cascades induce multiple responses, among which is altered gene transcription [3,4].

We previously demonstrated that mitogen-activated protein kinase-activated protein kinase 3 (MK3/3pK/MAPKAPK3) binds Polycomb repressive complex 1 (PRC1), via the self-association motif (SAM)-domain of PHC [5]. Polycomb Group and Trithorax Group proteins maintain transcriptionally repressed and activated epigenetic states respectively and as such are part of an important cellular transcriptional memory system. Although PRC1-mediated transcriptional repression has long been considered a stable repressive state, increasing evidence indicates that PRC1 repression is a dynamic process [6,7]. Genome-wide chromatin association studies have revealed changes in Polycomb/chromatin distribution and transcriptional reprogramming during lineage commitment and differentiation [8-10]. As M/SAPKs and MKs also play important roles in differentiation, development and cell proliferation, the physical association of PRC1 and MK3 suggests a functionally relevant connection. We have previously established that PRC1/chromatin dissociation correlates with their phosphorylation status during cell cycle progression [11]. Subsequently, we showed that acute mitogenic and stress signaling also results in PRC1/chromatin dissociation [5]. The molecular mechanisms that underlie this dynamic relocation of PRC1 and the exact role of MK3 in signaling to chromatin remained unknown. We here hypothesized that M/SAPK-MK signaling imposes a molecular mechanism by which cells regulate PRC1/chromatin association and PRC1 target gene expression in response to environmental cues. We used the previously identified PRC1 target gene *ATF3* to study PRC1-mediated transcriptional regulation [12]. *ATF3* is an immediate-early response gene (IEG) [13]; relevantly, ERK and MK have been implicated in IEG activation [14]. Therefore, mitogen-induced *ATF3* activation represents a suitable model to study the biological relevance of ERK/MK3/PRC1 signaling. Our current data establish that *ATF3* expression is dynamically controlled by MAPK/MK/PRC1, and identify important players in an epigenetic switch-module in response to changes in the cellular microenvironment.

## Results and Discussion

### M/SAPK signaling controls PRC1 target gene transcription

**Figure 1:**
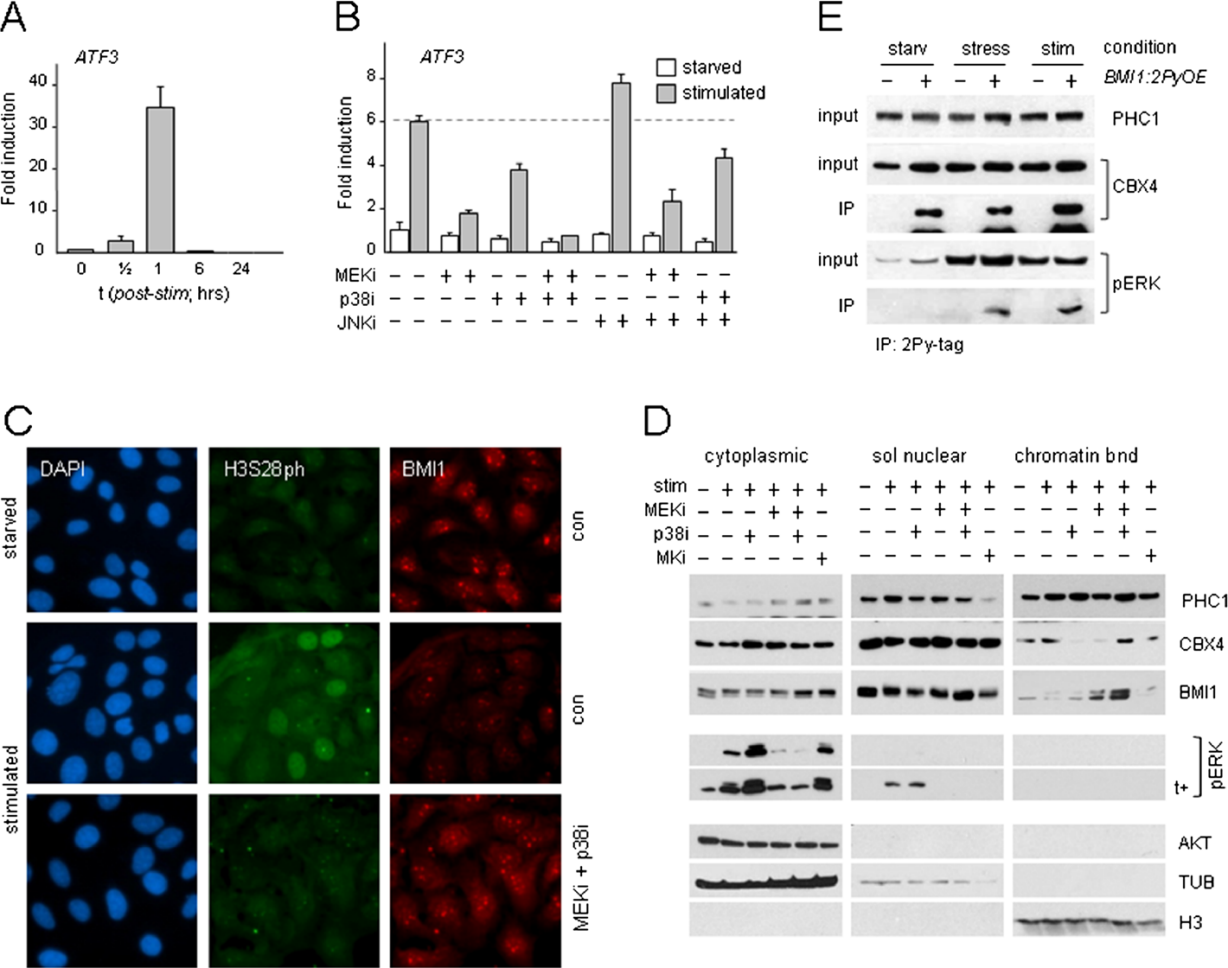
**PRC1 target gene expression is controlled by M/SAPKs.(A**,**B)** PRC1 target gene *ATF3* expression (mRNA) in mitogen-stimulated (FCS/TPA) TIG3 cells, in the absence or presence of kinase inhibitors; data ± SD. **(C)** H3S28ph and BMI1 staining in starved or mitogen-stimulated U2-OS cells; DAPI: nuclear counterstain. **(D)** Subcellular PRC1 protein distribution in starved and stimulated U2-OS cells; nuclear soluble and chromatin-bound fractions were proportionally loaded; nuclear fractions correspond to approximately three to four cytoplasmic equivalents. Cytoplasmic and nuclear fractions were always loaded on the same gel for direct quantitative comparison (corresponding sections are shown separately; representative experiment shown). AKT, TUB, H3: fractioning and loading controls (quantification chromatin-bound BMI1: compare Additional file 1: Figure S1C).**(E)** Induced interaction of pERK and BMI1 in response to mitogens (stim) or selenite (stress) in U2-OS cells; loading controls: PRC1 proteins CBX4 and PHC1; PCR1 interaction confirmed by BMI1/CBX4 co-immunoprecipitation (co-IP); +/−: transfected *BMI1-2Py* cDNA.

To determine how M/SAPKs are implicated in Polycomb-mediated repression, transcription of the PRC1 target gene *ATF3* was measured in the presence or absence of kinase inhibitors. All cells were serum starved (G0/G1-arrest) prior to stimulation throughout this study, to avoid interference by late S/G2-associated PRC1/chromatin dissociation [11]. ERK, P38 and jun N-terminal kinase (JNK) are all phosphorylated in response to mitogenic stimulation (Additional file 1: Figure S1A). Consistent with immediate-early response kinetics, *ATF3* mRNA levels rapidly increase and peak at 60 minutes after stimulation (Figure 1A). P38 inhibition (p38i) and MEK/ERK inhibition (MEKi) both substantially and reproducibly reduce *ATF3* induction; the effect of ERK inhibition is consistently stronger than p38i (± 3- to 4-fold versus ± 1.5- to 2-fold, respectively); combining both inhibitors blocks *ATF3* expression completely; JNK inhibition (JNKi) slightly enhances *ATF3* induction (Figure 1B). Various PRC1 proteins cluster to pericentromeric heterochromatin-associated Polycomb Group nuclear bodies (PcG-NB) in several cancer cell lines [11,15,16]. Using PcG-NB/ chromatin dissociation in U2-OS cells as a read-out for PRC1/chromatin binding [5,11], we find that PcG-NB/chromatin dissociation correlates well with increased histone H3 phosphorylation at Ser28 (H3S28ph) in mitogen-stimulated control cells, in concordance with previous observations [5], and that combined MEKi/p38i blocks both events (Figure 1C). H3S28 is phosphorylated by numerous triggers and MAPKAPK-family kinases, including RSKs and MSKs [17,18]. Of note, immediate-early response genes (IEG) are controlled by MKs and local H3S28 phosphorylation [14,19]. In the context of mitogenic stimulation, we find that P38i alone can block H3S28ph and chromatin dissociation of the PRC1 protein BMI1, whereas BMI1/chromatin binding is largely retained when ERK signaling is interrupted (MEKi), without affecting H3S28ph (Additional file 1: Figure S1B). These results support the notion that P38 targets H3S28 and that ERK signaling may target PRC1/chromatin association, and possibly PRC1 protein function and/or interaction. To examine the effect of mitogenic stimulation on subnuclear distribution of PRC1 proteins (in the presence or absence of kinase inhibitors), cells were differentially extracted to yield cytoplasmic, soluble nuclear and chromatin-bound fractions. We previously found that the PRC1 protein PHC1 undergoes a global chromatin redistribution in response to cell stress in contrast to some other PRC1 members [5]; as such, determination of total (that is, PcG-NB and global) PRC1 protein/chromatin association is pivotal. Of note: mitogenic stimulation differentially affects global PHC1/, CBX4/ (both increased) and BMI1/chromatin association (decreased), suggesting differential regulation of PRC1 members by M/SAPKs. Consistent with this, single inhibitor treatment (p38i or MEKi) clearly affects chromatin association of PRC1 proteins in distinct ways (Figure 1D). Combined inhibitor treatment of stimulated cells shows that more BMI1 remains chromatin-associated, consistent with the immunofluorescence (IF) analysis (Figure 1D,C, Additional file 1: Figure S1C); also CBX4/ and PHC1/chromatin binding are increased under p38i/MEKi conditions. The fluctuation of CBX4 levels suggests a direct involvement of phosphorylation in relation to chromatin association. Besides differential phosphoregulation of PRC1 proteins by M/SAPKs, the above findings predicted an interaction between MAPK and PRC1, downstream of mitogenic signaling. Indeed, co-immunoprecipitation analyses confirm a signaling-induced interaction between pERK and PRC1 complexes (Figure 1E). The PRC1/pERK interaction is counteracted by P38 signaling, as more pERK co-precipitates in a PRC1-directed IP in p38i cells (Additional file 1: Figure S1D, compare Figure 1D), in support of cross-talk between kinases at multiple levels. The combined data demonstrate that mitogenic signaling, via both activated P38 and ERK, converges at the level of PRC1/chromatin regulation and target-gene activation, and establish a relevant functional interaction between these signal transducers and PRC1.

### Transcription correlates with PRC1 dissociation, not loss of H3K27me3

**Figure 2:**
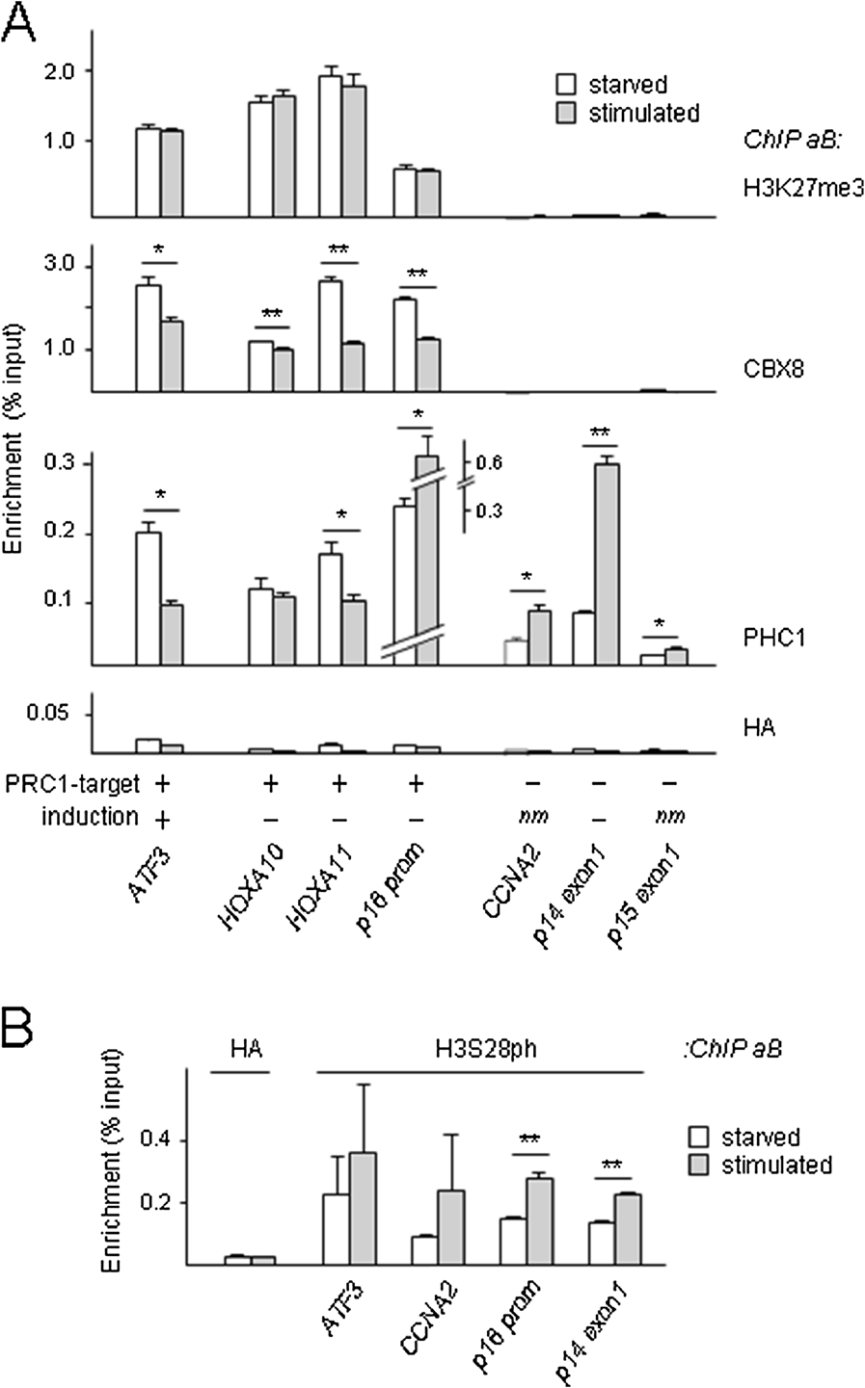
**PRC1/chromatin dissociation, not loss of H3K27me3, correlates with transcription. (A**,**B)** Chromatin immuno-precipitation (ChIP) analysis of PRC1 protein binding, histone H3K27me3 **(A)** and H3S28ph marking **(B)** at known PRC1 target and non-target loci in G1-arrested (starved) and mitogen-stimulated TIG3 cells. ChIP antisera are indicated in the figures (ChIP antibodies (aB)). (Statistical significance: *P < 0.05, **P < 0.01; *t*-test.

We next studied changes in PRC1 (CBX8, PHC1) chromatin occupation and H3K27me3 marks at established PRC1 target genes [12]. CBX8 and PHC1 show distinct chromatin-occupation profiles: whereas CBX8 is exclusively enriched at PRC1 target loci and is released upon serum stimulation at most targets, PHC1 appears present also at non-target genes and shows opposing dissociation dynamics at targets (decreased; *ATF3; HOXA11*) and non-targets (increased; *p15 exon1, CCNA2, p14*^*ARF*^*exon1*) upon stimulation (Figure 2A). The observed changes in PHC1 occupation are consistent with enhanced chromatin association in response to mitogens (compare Figure 1D). We find a clear correlation between simultaneous chromatin dissociation of CBX8 and PHC1, and activation of expression at the *ATF3* locus (Figure 2A, Additional file 2: Figure S2A). As CBX8 levels remain unaltered throughout the duration of the experiment, this does not account for the reduced CBX8/chromatin association (Additional file 2: Figure S2B). In contrast to CBX8, PHC1 increases at all non-induced loci (Figure 2A, Additional file 2: Figure S2A). The contrasting chromatin-binding dynamics of CBX8 and PHC1 in response to signaling are in good agreement with our previous observation that PHC1/chromatin binding appears to increase globally in the context of cell cycle progression or cell stress, whereas detection of other PRC1 members in PcG-NB (like BMI1, CBX4 and RNF2) is reduced [5,11]. Of note, PHC/PRC1-core complex association was reported to be relatively weak [20]. In addition, purified PHC1 is known to reside in non-PRC complexes [21]. Combined, these results indicate that PHC1 occupation occurs without the need for simultaneous CBX8 binding, and further supports the notion of differential regulation of PRC1-complex members. The observation that both CBX8 and PHC1 removal correlate with *ATF3* expression, whereas PHC1 is retained/increased at the CDKN2A/INK4A locus (Figure 2A, Additional file 2: Figure S2A) suggests that multiple/all PRC1-core complex members may need to dissociate at crucial regulatory regions to allow active transcription. It is conceivable that signaling through M(S)APK/MK3/PRC1 changes the local epigenomic state from a repressed/poised state [12,22,23] to a ‘de-repressed’ and eventually fully transcriptionally active state through progressive loss of PRC1 members.

Consistent with a global mitogen-induced increase of H3S28ph, ChIP analysis shows increased H3S28ph enrichment at PRC1 targets as well as non-targets; this epigenetic change occurs independent of transcriptional induction (Figure 2B, Additional file 2: Figure S2A). Remarkably, the unaltered H3K27me3 occupation at the *ATF3* promoter suggests that transcriptional activation of PRC1 target genes occurs without concomitant loss of H3K27me3 (Figure 2A). Thus, although PcG-NB/chromatin dissociation and H3S28ph correlate well [5,24,25], H3S28ph by itself does not determine transcriptional status. In addition, our findings argue that local maintenance of H3K27me3 does not obstruct transcriptional reactivation of PCR1 targets. The recently reported presence of H3K27me3 marks on active promoters supports this notion [26]. Whether or not the H3K27me3 mark needs to be removed at reactivated genes remains contradictory at this point [24-26], and may, besides on models and tools used, depend on the subgenic location of epigenetic modifications (possibly in conjunction with other modifications). Consistent with this idea, repressive H3K27me3 marking correlates best with transcription at loci that display ‘blanketing’-type H3K27me3 enrichment, that is, gene body-wide (downstream of the transcription start site (TSS)) [26]. It is conceivable that H3K27me3 promoter marking defines a specific class of response factors, which are dynamically controlled by rapid removal of PRC1 complexes. Thus far, our H3S28ph analyses suggest that H3S28ph, like CBX8 dissociation, is a global event; despite being essential for PRC1 target gene activation, it is clearly not the sole determining factor and as such is unlikely to specify regulation only at PRC1 target genes. Co-occurrence of methylphosphoryl modifications on adjacent histone lysines and serines was proposed to act as a binary epigenetic switch mechanism, by affecting chromobox-domain binding to histone-trimethyl marks [24,27-29]; Interestingly, also EZH2/chromatin dissociation at muscle-specific promoters during myogenesis appears driven by local H3K27 methyl/H3S28 phosphoryl modification [30,31]. The simultaneous detection of local H3S10ph and HP1 binding, however, seemingly challenges the strictness of the methylphosphoryl switch concept [32]. Irrespective of the exact mechanism, our combined molecular epigenetic analyses suggest PRC1 target gene activation does not require loss of H3K27me3 and point to chromatin dissociation of multiple PRC1 proteins as an important event in transcription initiation.

### MK3 negatively regulates ERK signaling

**Figure 3:**
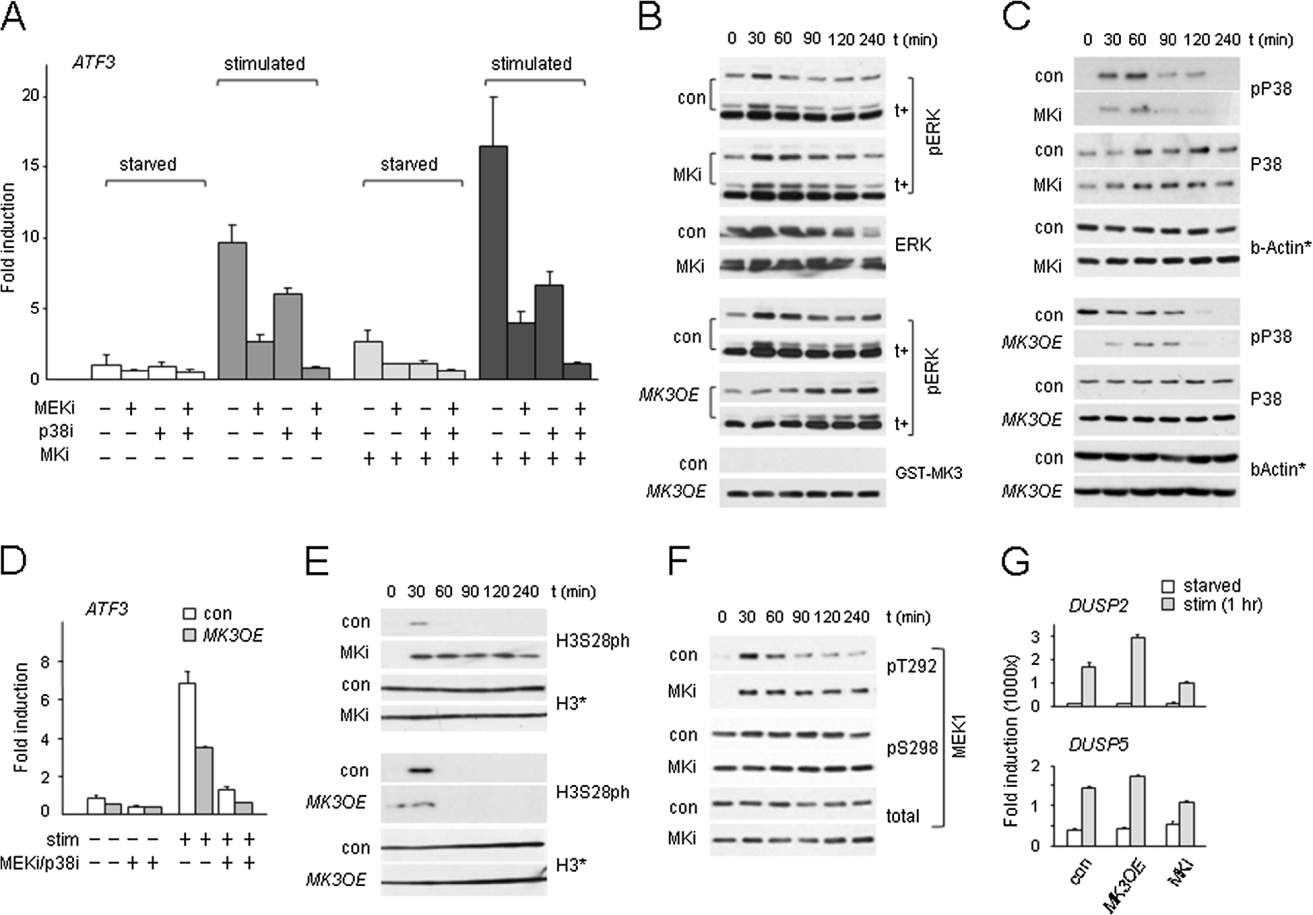
**MK3 is a negative regulator of ERK.(A,D)** mRNA expression of PRC1 target gene *ATF3* as a function of kinase inhibition **(A)** or *MK3OE***(D)** in TIG3 cells. **(B**,**C, E, F)** (p)ERK **(B)**, (p)P38 **(C)**, H3S28ph **(E)** and (p)MEK **(F)** levels in starved and stimulated U2-OS cells in relation to MK function; t (min): time post-stimulation in minutes; *: loading controls for corresponding panels; t+: longer exposure. Samples corresponding to control and experiment (*MK3OE* or MKi) were always loaded on the same gel to enable direct quantitative analysis (corresponding sections are shown separately; representative experiment shown; quantification pERK profiles: compare Additional file 3: Figure S3B). **(G)** Mitogen-induced *DUSP2* and *DUSP5*-mRNA in control, *MK3OE* and MKi TIG3 cells.

To determine the role of MK in PRC1 target gene regulation, we next studied the effects of MK inhibition or MK protein level modulation on *ATF3* gene expression in TIG3 and U2-OS cells. MK inhibition (MKi) releases basal repression of *ATF3* in starved MKi cells and allows for more robust *ATF3* mRNA induction; in contrast, treatment with MEKi or p38i, both suppress transcription (Figure 3A, Additional file 3: Figure S3A). Of note: MKi does not show an added effect on *ATF3* mRNA synthesis in combination with other single or multiple kinase inhibitors, suggesting that MKs exert their effect upstream of ERK and P38 signaling (Figure 3A). Thus, our data suggests a negative regulatory role for MKs in mitogenic signaling through MEK/ERK. In good agreement with this data, *MK*-deficient cells (by MKi or shRNA-mediated knockdown (*shMK*)) display sustained ERK1/2:T202/T204 phosphorylation (pERK; up to 90 to 120 minutes, compared to control cells) upon stimulation (Figure 3B, Additional file 3: Figure S3B,C). Conversely, *MK3* overexpression (*MK3OE*) reduces both basal and induced *ATF3* transcription, and, in combination with MEKi/p38i, further represses expression (Figure 3D). As predicted, *MK3OE* delays ERK phosphorylation, consistent with an inhibitory action of MK3 on ERK activity and reduced target-gene induction (Figure 3B,D, Additional file 3: Figure S3B). MK2 was recently found to stabilize P38 in a kinase domain-independent manner, that is*,* physical association of MK2 to P38 controls P38 stability [33]. Analogous with this report, we detect a rise in P38 levels in *MK3OE* cells versus reduced P38 levels in *shMK* cells; relevantly, P38 levels remain unaltered in MKi cells (Figure 3C, Additional file 3: Figure S3D). Contrary to pERK, P38:T180/T182 phosphorylation (pP38) is reduced in *MK3OE* cells as well as in *shMK* and MKi cells. Also JNK:T183/T185 phosphorylation (pJNK) is reduced by *shMK*, whereas *MK3OE* correlated with enhanced JNK levels (Additional file 3: Figure S3E,F). Although the full extent of factors and mechanisms controlling M/SAPK phosphorylation is not clear at this point, these findings are in keeping with multiple complex regulatory cross-interactions between MKs and M/SAPKs [3,34].

Consistent with our IF analyses (compare Figure 1C, Additional file 1: Figure S1B), H3S28 shows rapid and transitory phosphorylation kinetics in response to mitogens (Figure 3E). Relevantly, global H3S28ph is increased and sustained in MKi cells, whereas it is blunted in *MK3OE* cells (Figure 3)E in line with altered PRC1 target gene expression under these respective conditions (compare Figure 3A,D). This data demonstrates that H3S28 phosphorylation kinetics respond to MK3 activity, and support a regulatory role for MK3 in epigenetic modulation of cell responses to environmental stimuli.

To gain further insight into the molecular mechanisms by which MK3 controls M/SAPK activity, we studied MEK, an ERK effector kinase. MEKpT292 and MEKpS298 were examined, as these two phosphoresidues are part of a mechanism that controls MEK activity [35]. Like pERK, MEKpT292 is also sustained in MK-deficient cells, suggesting that MK3 controls phosphatase activity directed toward upstream kinases (Figure 3)F. Dual-specificity phosphatases (DUSPs) play important roles in feedback loops on phosphorylation cascades [36]. *DUSP2* and *DUSP5* induction meet the criteria of being dependent on mitogenic signaling and on MK, as *MK3OE* and MKi, enhance and decrease *DUSP* mRNA induction, respectively (Figure 3G). To validate the involvement of DUSPs in the negative regulatory network involving MK3, we used induction of EGR1 as a read-out for mitogen-induced ERK activity [37]. MKi cells display a stronger EGR1 induction (Additional file 4: Figure S4A), consistent with loss of negative regulation via ERK. To validate a role for phosphatase activity in regulation of the ERK/EGR1 response, cells were pretreated with orthovanadate, a generic DUSPs inhibitor (DUSPi). Enhanced EGR1 expression in the presence of DUSPi resembles that observed in MKi cells and supports a negative regulatory role for DUSPs in the MK3/ERK/EGR1 response (Additional file 4: Figure S4B); in line with reduced negative feedback in DUSPi cells, both pERK and pP38 are increased in response to mitogenic stimulation. Relevantly, also signaling-induced H3S28ph levels are enhanced significantly in the presence of DUSPi, in agreement with a functional role for DUSPs in epigenetic regulation (Additional file 4: Figure S4B). Relevantly, the reduced ERK phosphorylation by *MK3OE* is partially reversed by DUSP inhibition, suggesting that DUSP activity acts as part of a relay system in the regulatory feedback of MK3 to MEK/ERK (Additional file 4: Figure S4C). Combined, the above findings support a negative regulatory role for MKs in MEK/ERK signaling, at least in part, via induction and/or activation of phosphatases.

**Figure 4:**
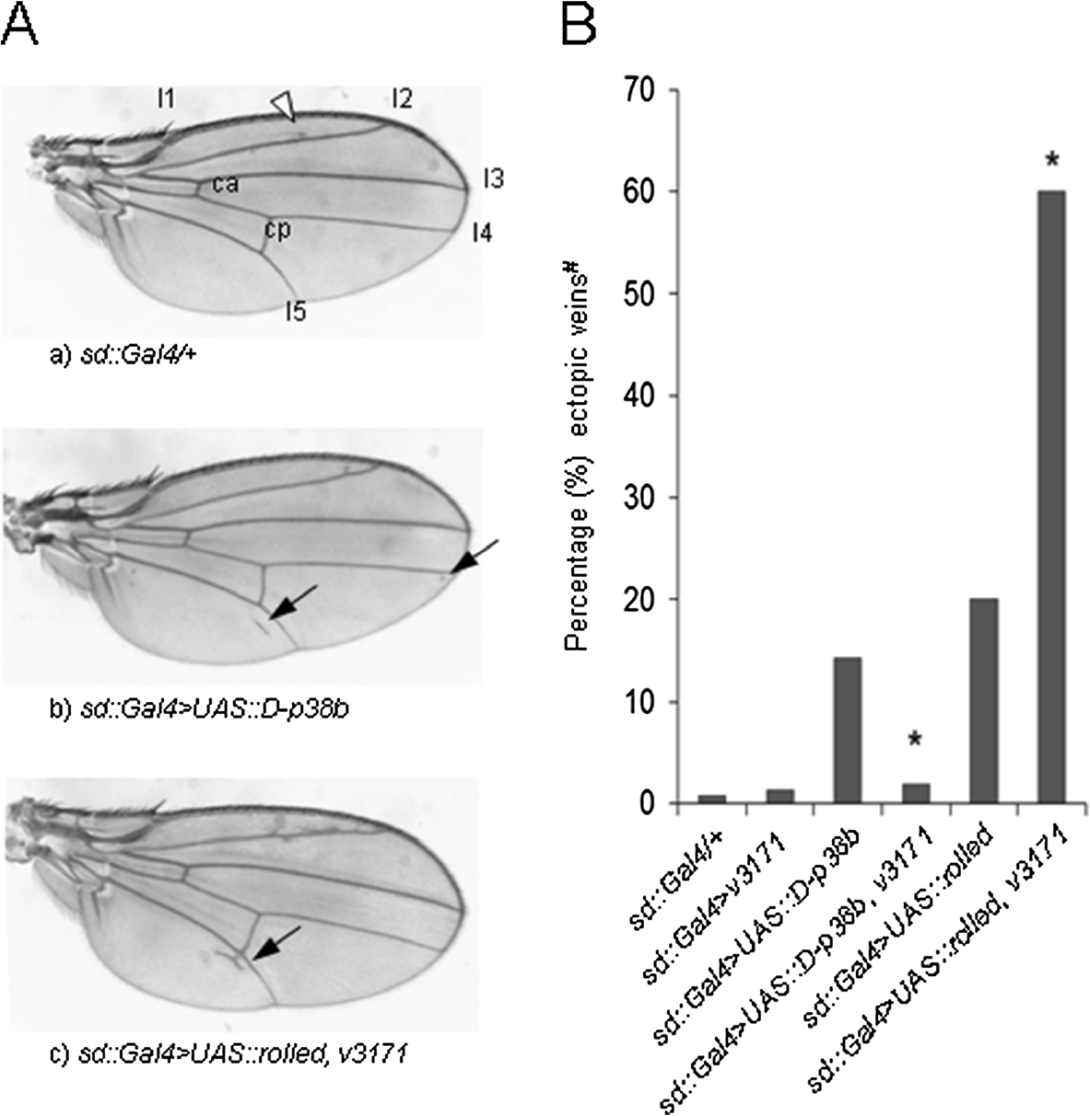
**Negative feedback by *dMK2* on *dERK/rolled* in ectopic wing vein development. (A, B)** Representative ectopic vein patterns (black arrows) in control **(a)** and transgenic lines **(b,c)**; percentages of flies with ectopic wing veins in different genetic contexts (indicated below graph; **B)**; **#**: *sd::Gal4* driver-induced ectopic veins nearby vein 2 (l2; white arrow head compare **A)**, were not scored; *: significantly different from corresponding genotype without *dMK2-LOF* (z-test; *P* < 0.001).

To obtain independent proof for the functional relationship between MKs and M/SAPKs, we examined kinase interactions in *Drosophila melanogaster*. We first reproduced a signaling-induced interaction between Ph-p (PHC1/2 ortholog) and dMK2 (the only *MK* ortholog in *D. mel*) in cultured insect cells (Additional file 5: Figure S5A). We next exploited *Drosophila* wing vein patterning to probe for M/SAPK-MK interaction *in vivo*. Wing vein formation in the fruit fly is dependent on MAPKs [38-40]. As expected, gain-of-function (GOF) lines for *dERK/rolled* (*sd::Gal4 > UAS::rolled; D.mel* ERK ortholog) show ectopic wing veins during wing imaginal disc development (Figure 4A,B.) We here report that flies overexpressing *D-p38b* (*sd::Gal4 > UAS::D-p38b;* one of the *D.mel* P38 orthologs) also display enhanced ectopic vein formation (Figure 4A,B). Hence the ectopic vein phenotype can be used to gauge the effects of MK loss-of-function (LOF) on ERK/P38 pathway activity. To determine the effect of dMK2 on dERK and D-p38b, we crossed a transgenic RNAi-mediated *dMK2*-knockdown line (*v3171*) to *dERK*- and *D-p38b*-GOF lines (see: Methods section, Additional file 5: Figure S5B). *dMK2*-LOF enhances the *dERK*-GOF ectopic vein phenotype (Figure 4)A,B. In contrast, *dMK2*-LOF suppresses the ectopic vein phenotype induced by *D-p38b*-GOF (Figure 4)A,B. Importantly, this data is fully congruent with our *in vitro* findings in mammalian cells and supports the existence of a complex genetic network between MAPKs, SAPKs and MKs. In conclusion, our *in vivo* experiments substantiate a negative regulatory role of *dMK2* on *dERK*-signaling in wing vein development.

## Conclusions

**Figure 5:**
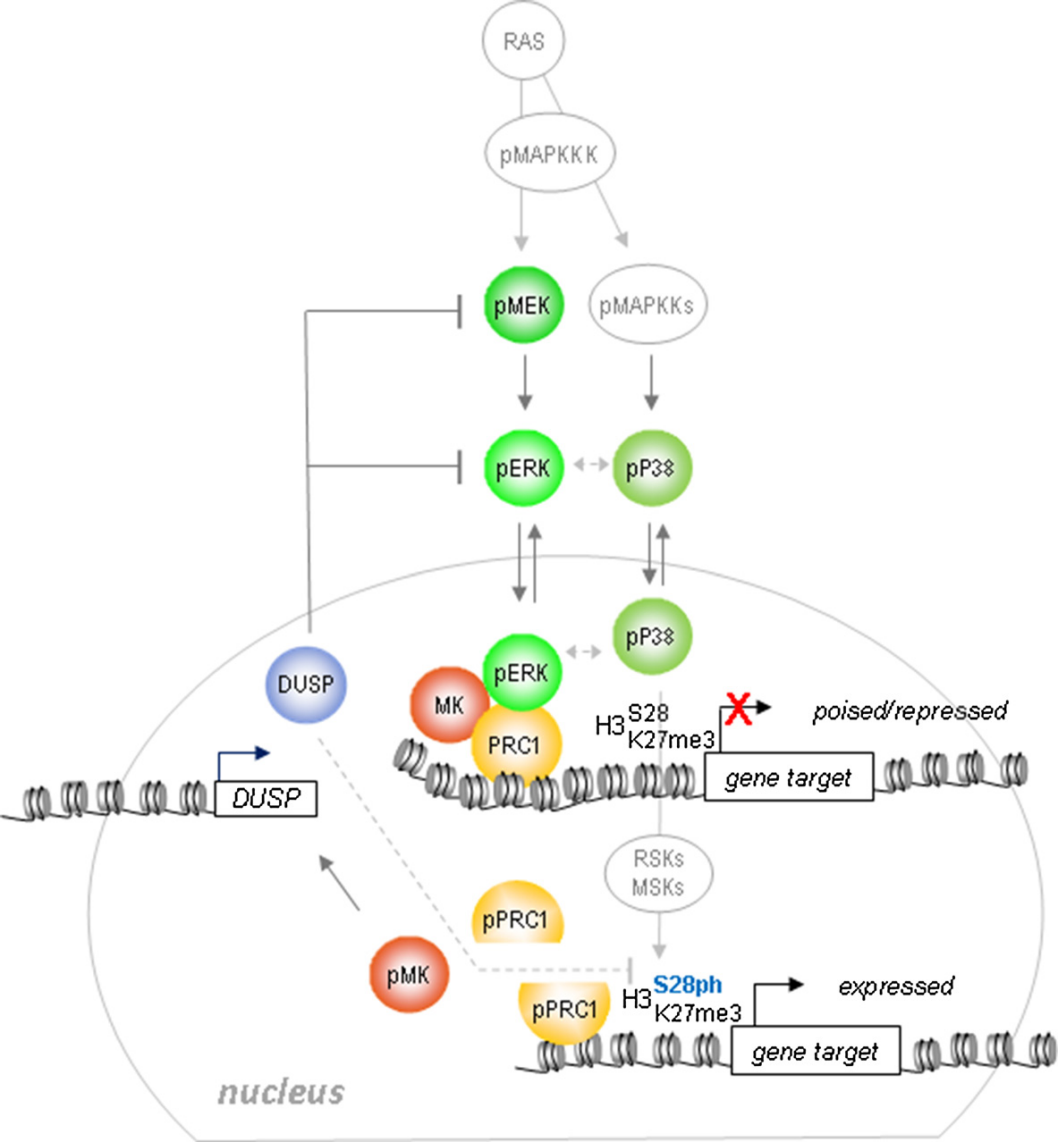
**Model for MAPK/MK regulation of PRC1 target genes.** The PRC1/MK/ERK module represents a molecular switch mechanism: fine-tuning of converging P38 and ERK signals at the chromatin level triggers a potential methylphosphoryl switching module and a signaling-dependent disruption of the PRC1/chromatin association that permits gene activation. Transient H3S28ph, but not removal of H3K27me3, accompanies gene expression. Concomitant MK activation initiates DUSP-dependent negative regulatory feedback on MEK/ERK (and possibly also on H3S28ph; dashed lines reflect potential functional relationships).

In summary, we here identify and carefully characterize a number of actors in PRC1-dependent gene regulation. Signaling through P38 has direct implications for local chromatin modulation (that is, H3S28 phosphorylation). We propose that ERK/PRC1/MK3 acts as a molecular dimmer switch that enables rapid and reversible transcriptional activation of *ATF3*. This mechanism defines negative feedback on Polycomb-mediated repression as an integral process in the regulation of appropriate cellular responses to environmental changes (Figure 5). Whereas part of the responses appears global, like H3S28 phosphorylation and CBX dissociation, the presence of PRC1/MK3 likely contributes to chromatin targeting of pERK and provides specific local regulation. PRC-complex members are emerging as proteins harboring many different functions and binding partners [7]. We recently categorized ± 120 known phosphorylation sites on a limited number of PRC proteins; many of these sites are predicted M/SAPK target sites and are likely to cooperate in gene regulation [41-44]. Although some phosphosites have been linked to PRC protein function [45-49], the biological relevance of the vast majority of these phosphorylation events is currently unknown. Our findings pave the way for detailed mutational analysis of the functional consequences of post-translational modification of Polycomb proteins. The findings presented herein provide novel insight into differential regulation of PRC1 members in response to signaling and add to the growing recognition that PRC1-mediated repression is a highly dynamic process.

## Methods

### Cell culture, viral infections

Human U2-OS osteosarcoma cells and TIG3 primary human fibroblasts expressing the murine ecotropic receptor were kindly provided by Dr. D. Shvarts (Utrecht Medical Center, Utrecht, The Netherlands) and Dr. D. Peeper (Netherlands Cancer Institute (NKI), Amsterdam, The Netherlands), respectively. Expression vectors encoding the murine ecotropic receptor were a courtesy of Dr. R. Bernards (NKI, Amsterdam, The Netherlands). All human cell lines were cultured under standard conditions in DMEM containing 10% fetal calf serum (FCS). Retroviral expression vectors were used to maximize percentages of expressing cells and to minimize integration effects. Production of infectious viral particles was carried out as described previously [50]. Briefly, ecotropic retroviral supernatants were produced by transfection of producer cells using calcium-phosphate co-precipitation. Forty to forty-eight hours post-transfection, the supernatants were harvested, filtered and stored at −80°C until further use. Viral titers were sufficiently high to achieve near 100% infection. Cells were transduced with retrovirus in the presence of 4 μg/ml polybrene (Sigma-Aldrich, St. Louis, MO, USA) at around 25% confluency for six to eight hours and then allowed to recover for forty-eight hours on fresh medium before selection pressure was applied. Transduced cells were grown for ± 1 week on 1 to 4 μg/ml puromycin (Sigma) preceding experiments. Expression of all plasmid constructs was verified by immunoblotting.

Cells were serum-starved at 0.1% FCS for 48 hours. Mitogenic stimulation was achieved by supplementing 15% FCS/100 ng/ml tetradecanoylphorbol acetate (TPA; Sigma) for 45 minutes or as indicated. Cells were pretreated with kinase inhibitors: 30 minutes 10 μM MEK inhibitor (U0126; Promega, Madison, WI, USA), 30 minutes 10 μM MK inhibitor (MK2a Inhibitor; Calbiochem/Merck, Darmstadt, Germany), 30 minutes 20 μM p38 inhibitor (SB202190; Calbiochem), 30 minutes 25 μM JNK inhibitor (SP600125; Biomol, Plymouth Meeting, PA, USA), 30 minutes 85 μM etoposide (ETP; Calbiochem). MK2a was confirmed to inhibit MK3 (data not shown). Cells were preincubated with sodium orthovanadate for 30 minutes prior to mitogenic stimulation at indicated concentration; control cells were incubated with solvent. Sodium selenite (Sigma) -treated cells (90 minutes 0.2 mM) were used as a positive control. *Drosophila* S2 cells were cultured at 25°C in Schneider medium with 10% FCS. The day before transfection, 5.10^6^ cells were transferred in medium with 0.1% FCS. For transfection, 2 μg of plasmid DNA were mixed with Effecten™ transfection reagent (Qiagen, Hilden, Germany) according to manufacturer’s instructions (1/10 DNA-Effecten™ ratio). Forty-eight hours later, cells were either untreated, stimulated with 10% FCS/100 ng/ml TPA or 0.5 mM sodium meta-arsenite for two hours at 25°C. For immunoprecipitation (IP) experiments, plasmids Act::Myc-Ph and Act::EGFP-MAPKAP were co-transfected into S2 Drosophila cells grown in 0.1% FCS. IPs were performed as described (Mouchel-Vielh *et al*., 2011); IP antibodies are listed in Additional file 6: Table S1.

### Expression constructs

Retroviral vectors (pBMN-LZRS.ires.GFP, pBMN-LZRS.ires.NEO) expressing murine BMI1-2Py or GST-MK3 have been described elsewhere [5,11]. Retroviral systems were used as published [51,52]. RNA-interfering MK3 sequences were cloned into stable shRNA vectors [53]; targeting sequences: Additional file 7: Table S2.

The *dMK2* cDNA was amplified from clone SD05481 (Drosophila Genomics Resource Center; for oligonucleotides: see Additional file 7: Table S2). The resulting amplicon was introduced into pENTR/D-TOPO™ (Invitrogen, Carlsbad, CA, USA), then transferred through LR-recombination into T. Murphy’s vectors *pAWG* (https://dgrc.cgb.indiana.edu/vectors). Similarly, a cDNA corresponding to the C-terminal part of *Drosophila* Polyhomeotic-proximal gene (*Ph* or *Ph-p*) was amplified, introduced in pENTR/D-TOPO™, and subsequently transferred in *pAMW* (for oligonucleotides: see Additional file 7: Table S2) [54]. *Ph* sequences in the *Act::myc-Ph* construct endoded the 346 C-terminal amino acids of the Ph-proximal protein (GenPept qualifiers: NP_476871.2 GI:24639272) and included the SAM domain through which MK3 and PHC1/2 interact [5].

### Chromatin immunoprecipitation (ChIP) assays

ChIPs on primary human fibroblasts were performed and analyzed essentially as described [12]. Cells were fixed for 10 minutes in 1% formaldehyde/phosphate buffered saline (PBS) and stopped by 5 minutes incubation in glycine (final concentration: 0.125 M). Fixed cells were washed twice with PBS and harvested in SDS buffer (50 mM Tris at pH 8.1, 100 mM NaCl, 0.5% SDS, and 5 mM EDTA), supplemented with protease inhibitors (aprotinin, antipain and leupeptin all at 5 μg/ml and 1 mM PMSF). Cells were pelleted by centrifugation, and suspended in IP buffer (100 mM Tris at pH 8.6, 100 mM NaCl, 0.3% SDS, 1.7% Triton X-100, and 5 mM EDTA), containing protease inhibitors. Cells were disrupted by sonication, yielding genomic DNA fragments with a bulk size of 200 to 500 bp. For each IP, 1 ml of lysate was precleared by adding 35 μl of blocked protein A beads (Protein A-Sepharose/CL-4B (GE Healthcare, Piscataway, NJ, USA); 0.5 mg/ml fatty acid-free BSA (Sigma); and 0.2 mg/ml herring sperm DNA in TE buffer), followed by centrifugation. 10 μl aliquots of precleared suspension were put aside as input DNA and kept at 4°C. Samples were immunoprecipitated overnight at 4°C. HA antiserum was used as negative control. Immune complexes were recovered by adding 40 μl of blocked protein A or G beads (GE Healthcare) and incubated for 4 hours at 4°C. Beads were washed three times in 1 ml of mixed micelle buffer (20 mM Tris at pH 8.1, 150 mM NaCl, 0.2% SDS, 1% Triton X-100, 5 mM EDTA, and 5% w/v sucrose), twice in 1 ml of buffer 500 (50 mM HEPES at pH 7.5, 1% Triton X-100, 1 mM EDTA, and 0.1% w/v sodium deoxycholate), twice in 1 ml of LiCl detergent wash buffer (10 mM Tris at pH 8.0, 1 mM EDTA, 0.5% sodium deoxycholate, 0.5% NP-40, and 250 mM LiCl), and once in 1 ml TE buffer. Immune complexes were eluted from beads in 250 μl elution buffer (1% SDS and 0.1 M NaHCO3) for 2 hours at 65°C (continuous shaking at 1000 rpm), and after centrifugation, supernatants were collected. 250 μl elution buffer was added to input DNA samples and these were processed in parallel with eluted samples. Cross-links were reversed overnight at 65°C, followed by 2 hours digestion with RNase A at 37°C and 2 hours proteinase K (0.2 μg/μl) at 55°C. DNA fragments were recovered using QIAquick PCR purification columns, according to manufacturer’s instructions (Qiagen, Hilden, Germany). Samples were eluted in 75 μl EB buffer and then diluted 1:5 in TE buffer. Immunoprecipitated DNA was quantified by real-time PCR (for ChIP antisera, primers: see Additional file 6: Tables S1, Additional file 7: Table S2). Each experiment was performed in triplicate. Results of one representative experiment are shown.

### RNA isolation, cDNA synthesis, real-time (RT) PCR analysis

For RT-PCR analysis, total RNA was isolated using Tri Reagent (Sigma) according to the manufacturer’s protocol. Quantity and quality of the RNA were determined by 260/280 nm and 260/230 nm absorbance measurements, respectively, using the Nanodrop (Witec AG, Luzern, Switzerland). Total RNA (1 μg) for each sample/replicate was converted into first strand cDNA using the iScript™ cDNA synthesis kit (Bio-Rad, Hercules, CA, USA) according to the manufacturer’s instructions. Gene expression was determined by RT-PCR using the MyiQ™ thermal cycler (Bio-Rad) in combination with the IQ5 version 2.1 software (Bio-Rad). RT-PCR was performed on 25 ng of cDNA using the qPCR iQ™ Custom SYBR™ Green Supermix with fluorescein (Bio-Rad) and 300nM primer in 96-well plates (Bio-Rad). For each primer pair a standard curve was generated with a serial dilution of a cDNA pool. RT-PCR data was analyzed according to the relative standard curve method. All values were normalized to either *beta-Actin* (Additional file 2: Figure S2) or *cyclophillin A* (Figures 1,3, Additional file 1: Figure S1, Additional file 3: Figure S3). The control condition is used as a reference. Primer sets for the selected genes were developed with Primer Express version 2.0 (Applied Biosystems, Foster City, CA, USA) using default settings (Additional file 7: Table S2).

### Immunofluorescence

Cells were grown on 6- or 12-well culture plates (Greiner Bio-One, Alphen aan de Rijn, The Netherlands) to ± 60 to 80% confluency, pretreated when applicable, washed twice with PBS, and either fixed for 15 minutes in 2% formaldehyde/PBS at room temperature (RT) followed by a 15 minutes incubation in chilled 100% methanol (MetOH) at −20°C, or directly fixed in 100% MetOH for 15 minutes at −20°C. Fixed plates were stored at 4°C in 70% ethanol (EtOH) or directly washed three times with PBS and used for immunocytochemistry (IC). For detection of PRC1 proteins and histone modifications, cells were first permeabilized for 10 minutes at RT in 0.2% Triton X-100 (TrX) in PBS. After extensive washing in PBS/0.02% TrX, cells were incubated with primary antibody (Additional file 6: Table S1) for 1.5 to 2.5 hours in a prewarmed humidified chamber at 37°C, washed five times in PBS/0.02% TrX and incubated with fluorescently labeled secondary antibody for 60 minutes at 37°C. 4′-6-diamidino-2-phenylindole (DAPI) was co-incubated with secondary conjugated antibodies to counterstain cell nuclei. Plates were washed in PBS/0.02% TrX, rinsed in PBS and subsequently dehydrated: 1 minute in 70% EtOH, two times 1 minute in 100% EtOH and air-dried. Cells were mounted in Vectashield (Vector Laboratories, Inc. Burlingame, CA, USA), analyzed using a Nikon TE200 Eclipse fluorescence microscope and photographed using a Nikon DXM1200 digital camera in combination with NIS-Elements 3.0 imaging software. All antibodies were diluted in blocking buffer (1% BSA, 5% FCS, 5% normal goat serum (NGS), in PBS/0.02% TrX). Secondary antisera used were goat-anti-mouse Texas Red™ (TXRD; 1:100; Southern Biotech, Birmingham, LA, USA) and goat anti-rabbit fluorescein isothiocyanate (FITC; 1:100; Southern Biotech), to detect monoclonal and polyclonal primary antibodies respectively.

### Protein isolation, differential extraction, immunoprecipitation (IP), immunoblotting (IB)

Protein extraction and immunoblotting (IB) were performed and analyzed as described previously with minor adjustments [2,5]. For extraction, cells were washed twice with cold PBS and lysed in RIPA buffer (50 mM Tris at pH 8.0, 150 mM NaCl, 0.1% SDS, 5 mM EDTA, 0.5% w/v sodium deoxycholate, and 1% NP-40) supplemented with protease and phosphatase inhibitors (5 mM benzamidine, 5 μg/ml antipain, 5 μg/ml leupeptin, 5 μg/μL aprotinin, 1 mM sodium orthovanadate, 10 mM sodium fluoride, 10 mM pyrophosphate, 10 mM ß-glycerophosphate, 0.5 mM DTT, and 1 mM PMSF). Lysates were subjected to two freeze-thaw cycles in liquid nitrogen, followed by sonication on ice with a probe sonicator (Soniprep 150; MSE, London, UK) for 12 cycli (1 sec ON, 1 sec OFF) with amplitude 5. After 10 minutes centrifugation at 13200 rpm (4°C; Eppendorf centrifuge), the supernatant was transferred to a fresh tube and protein concentration was determined using a BCA protein assay kit (Pierce/Thermo Fisher Scientific, Rockford, IL, USA) according to the manufacturer’s protocols on a Benchmark 550 microplate reader (Bio-Rad).

For differential extraction, cells were washed two times with cold PBS and scraped in lysis buffer (Tris HCl at pH 7.5, 150 mM NaCl, 0.5% Triton X-100, 1 mM EDTA; supplemented with inhibitors). After 30 minutes incubation on ice, nuclei are collected in the pellet by centrifugation (8000 rpm; 4°C) the supernatant is the cytoplasmic fraction. Nuclei are washed in lysis buffer and suspended in ELB buffer and incubated on ice for 10 minutes. Nuclear soluble (supernatant) and chromatin-bound fractions (pellet) are separated by centrifugation (13200 rpm; 4°C). After an additional wash in ELB buffer, the pellets were suspended in ELB buffer and sonicated.

For IP, cells were either cross-linked followed by cell lysis, or cells were lysed directly in ELB buffer (50 mM HEPES at pH 7.0, 250 mM NaCl, 5 mM EDTA, and 0.1% NP-40) supplemented with 5 mM benzamidine, 5 μg/ml antipain, 5 μg/ml leupeptin, 5 μg/ml aprotinin, 1 mM sodium vanadate, 10 mM sodium fluoride, 10 mM pyrophosphate, 10 mM ß-glycerophosphate, 0.5 mM DTT, and 1 mM PMSF. For cross-linking, cells were incubated in 1% formaldehyde (in PBS) for 10 minutes at RT, fixation was stopped by addition of 2 M glycin to a final concentration of 0.125 M. After 5 minutes incubation, cells were washed twice in cold PBS and lysed in ELB buffer. IP was carried out as described [11,15]. Briefly, extracts were sonicated on ice, centrifuged for 10 minutes at 4°C at 13200 rpm and supernatants were transferred to a precooled tube; 10% of the supernatant was taken as input and stored at −80°C. Appropriate amount of antiserum (Additional file 6: Table S1) was added and tubes were rotated for 1 hour at 4°C on a spinning wheel. To precipitate immune complexes, washed protein G beads (Protein G Sepharose/4 Fast Flow; GE Healthcare) were added to the extracts and rotated for 3 to 4 hours at 4°C, followed by 3 minutes centrifugation at 3000 rpm at 4°C. Supernatant was collected as depleted fraction and stored at −80°C. Beads were washed four times in ELB buffer (with supplements) and stored dry at −80°C until IB analysis.

For IB, equal protein amounts were boiled in sample buffer for 5 minutes and loaded on 9 to 15% polyacrylamide gels. Following separation by SDS-PAGE, proteins were transferred onto polyvinylidene fluoride (PVDF) membranes (GE Healthcare). Ponceau S (Sigma) staining was used to check protein transfer. PVDF membranes were blocked with 3.4% non-fat dry milk (Protifar; Nutricia, Zoetermeer, the Netherlands) in PBS containing 0.1% Tween 20 (pH 7.5) for 1 hour at RT, followed by an overnight incubation at 4°C with the primary antibody (Additional file 6: Table S1; anti-CBX4 [55]). After extensive washing with PBS/0.2% Tween 20, membranes were probed with corresponding horseradish peroxidase-conjugated secondary antibodies for 1 hour at RT: goat anti-rat (7077; 1:2,000; Cell Signaling, Danvers, MA, USA), rat anti-mouse (P0260; 1:5,000; DAKO, Glostrup, Denmark) and donkey anti-rabbit (711035-152; 1:15,000; Jackson Lab, Bar Harbor, ME, USA), to detect monoclonal rat, monoclonal mouse and rabbit polyclonal primary antibodies respectively. Signals were detected on autoradiograms using enhanced chemoluminescence (ECL; Pierce). Intensity of the bands was quantified with Quantity One software (Bio-Rad).

### Drosophila lines

*Drosophila melanogaster* stocks and crosses were kept on standard media at 25°C. For crosses, five females were mated with five males; they were transferred each 48 hours in new tubes. The *w*^*1118*^ line was used as control line. The *UAS::rolled* line was a gift from Dr. K. Moses (University of Cambridge, Cambridge, UK); the *UAS::-p38b* was a gift from Dr. JM Gibert (University of Geneva, Geneva, Switzerland); *UAS::rolled* and *UAS::D-p38b* allow *dERK* and *-p38b* overexpression, respectively [38,56]. The *v3171* line (*w*^*1118*^*;* MAPk-Ak2^GD1597^) that downregulates *dMK2* by RNA interference, was purchased from the Vienna *Drosophila* RNAi Center (VDRC) [57]; it does not present any known off-target effects. Downregulation of *dMK2* in third instar larvae was verified by real-time (RT) PCR using primers located outside the *v3171* repeats (Additional file 5: Figure S5B) as described [41]. Results were normalized against rp49 or Spt6 (Additional file 7: Table S2). Transgene overexpression was achieved using the wing-specific *Gal4* transgenic driver *scalloped sd*^*29.1*^*(*called *sd::Gal4;* BL-8609 line; Bloomington *Drosophila* Stock Center (BDSC), Bloomington, IN, USA)). All the transgenic lines display Gal4-independent *mini-white* expression that allows tracing of transgene transmission. *dMK2*-LOF and *hMK3*-GOF transgenic flies were also crossed to mutant Polycomb lines to study a potential interaction of MK and PcG *in vivo*. Neither *hMK3-*GOF nor *dMK2-*LOF induce a discernable *sexcomb*-phenotype by themselves or in combination with established *Pc*^*1*^ and *Scm*^*D1*^ alleles in heterozygote crosses (data not shown). The most likely explanation for this lack of phenotype is that *dMK2* may not play a role in anteroposterior (AP) patterning. In support of the latter, AP-axis abnormalities have not been reported in single or double MK2/3 knockout mice [34].

## Competing interests

The authors declare that they have no competing interests.

## Authors’ contributions

PP, EM-V, YT, URR, FP, JWV conceived of the study design; PP, HN, EM-V, GvdA, FS, VD, CG, MA, FP, JWV performed the experiments and analysis; PP, FP, and JWV drafted the manuscript. All authors read and approved the final manuscript.

## Supplementary Material

Additional file 1 **Figure S1.** PRC1 target gene expression is controlled by ERK and P38. **(A)**. MAPK and SAPK phosphorylation in response to mitogenic stimulation (stim) in U2-OS cells; specificity of response was supported by distinctive phosphorylation profiles induced by two different stressors (ETP: etoposide, Se: selenite). **(B)** H3S28ph and PRC1 protein (BMI1) staining in G1-arrested (starved) or mitogen-stimulated U2-OS cells. **(C)** Quantification of chromatin-bound BMI1 levels (compare Figure 1D) in mitogen-stimulated cells pretreated with kinase inhibitors (indicated); BMI1 levels were normalized versus histone H3. **(D)** Interaction of pERK and BMI1; U2-OS/BMI2Py cells were stimulated with mitogen (stim) prior to IP; t+: longer exposure (IB: immunoblot; IP: immunoprecipitation). Parallel experiments with pP38 were inconclusive due to IB detection issues with applied antisera. Click here for file

Additional file 2 **Figure S2.** PRC1/chromatin dissociation, not loss of H3K27me3, correlates with transcription. **(A)** mRNA expression of PRC1 target and non-target genes in TIG3 cells upon 0, 45 and 90 min of mitogen-stimulation. **(B)** Mitogenic stimulation induces BMI phosphorylation (upper panel; pBMI1) in TIG3 cells; cellular BMI1 and CBX8 protein (lower panel) levels remain unchanged under these conditions in support of changed PRC1/chromatin association rather than loss of protein (for example, degradation). Click here for file

Additional file 3 **Figure S3.** MK3 is a negative regulator of ERK. **(A)** mRNA expression of PRC1 target gene *ATF3* as a function of kinase inhibition in TIG3 cells (data as in Figure 3A) and U2-OS cells. **(B)** Quantification of pERK profiles (compare Figure 3B) in control versus *MK3OE* cells (left panel) and in control versus MKi cells (right panel); pERK levels were normalized to beta Actin (b-Actin). **(C-F)** IB-analysis of (p)ERK (C), (p)P38 (D), (p)JNK (E) in resting or mitogen-stimulated (stim) control and MK-knockdown (*shMK*) cells and (F) in the context of *MK3OE*; t (min): time post-stimulation in minutes. Samples corresponding to control (con) and experiment (*MK3OE* or *shMK*) were always loaded on the same gel to enable direct quantitative comparison; *: loading controls for all corresponding panels; t+: longer exposure. Click here for file

Additional file 4 **Figure S4.** DUSP involvement in regulation of MK3/ERK. **(A)** IB analysis of ERG1 in resting or mitogen-stimulated (stim) control (con) and MK-inhibited (MKi) cells; t (min): time post-stimulation in minutes. Samples corresponding to control and experiment (*shMK*) were always loaded on the same gel to enable direct quantitative comparison. **(B)** Cells were pretreated with DUSP inhibitor (DUSPi; concentration indicated) prior to mitogenic stimulation. IB detection of proteins as indicated. **(C)** Cells were pretreated with 50 μM DUSPi prior to mitogen stimulation. Samples corresponding to control and experiment (*MK3OE*) were always loaded on the same gel (bottom panel) to enable direct quantitative comparison. Quantitative analysis of effects of DUSPi in control cells (con; left panel) and *MK3OE* cells (right panel); normalization pERK levels was done versus beta Actin (b-Actin). Click here for file

Additional file 5 **Figure S5.**PRC1/MK/ERK module represents a molecular switch mechanism. **(A)** Functional interaction between dMK2 and Polyhomeotic *in vitro*: stress signaling-induced interaction of EGFP-tagged dMK2 (*Drosophila* ortholog of MK2/3) and Myc-tagged Ph (*Drosophila* ortholog of PHC1/2) in S2 cells. The Myc-Ph sequences comprised the C-terminal SAM domain [54]. Conditions tested: starved, mitogen-stimulated (FCS/TPA) and stressed (arsenite). IgG represents IgG-heavy and -light chains. **(B)** Reduced dMK2 expression in third instar *v3171* larvae; normalization indicated. Click here for file

Additional file 6 **Table S1.**Antibodies used for ChIP, ICC, IB and IP. Click here for file

Additional file 7 **Table S2. P**rimer sequences and targeting sequences. Click here for file
