# Supplementary figures and images for: MK3 controls Polycomb target gene expression via negative feedback on ERK
Source: Epigenetics Chromatin. 2012 Aug 7;5:12. doi: 10.1186/1756-8935-5-12 (PMC3499388; doi:10.1186/1756-8935-5-12)

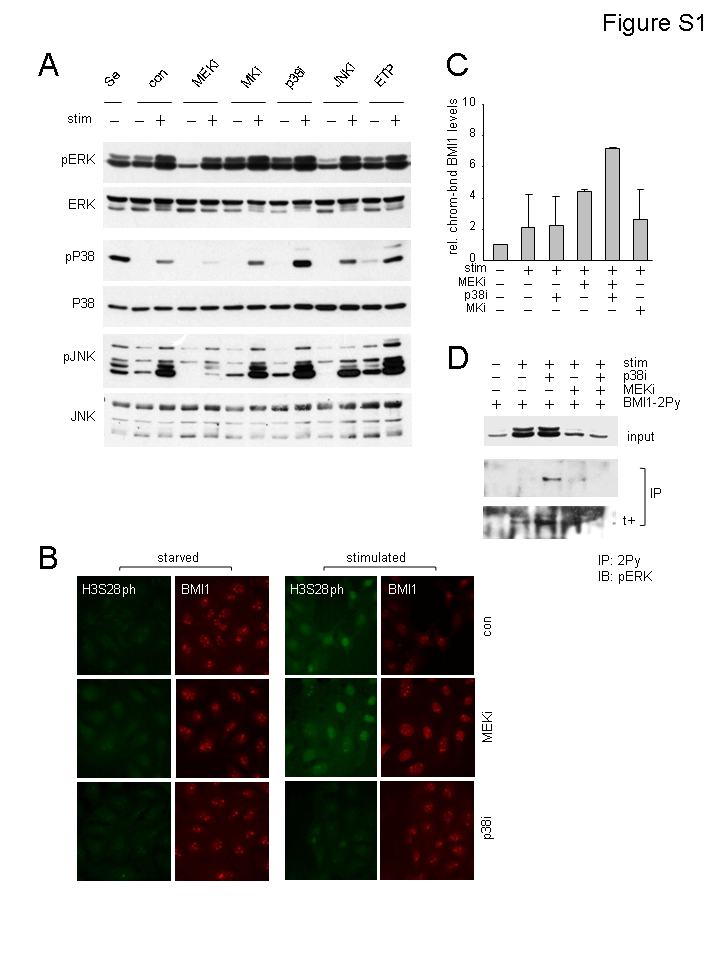

Supplement: Additional file 1 — Figure S1. PRC1 target gene expression is controlled by ERK and P38. (A). MAPK and SAPK phosphorylation in response to mitogenic stimulation (stim) in U2-OS cells; specificity of response was supported by distinctive phosphorylation profiles induced by two different stressors (ETP: etoposide, Se: selenite). (B) H3S28ph and PRC1 protein (BMI1) staining in G1-arrested (starved) or mitogen-stimulated U2-OS cells. (C) Quantification of chromatin-bound BMI1 levels (compare Figure 1D) in mitogen-stimulated cells pretreated with kinase inhibitors (indicated); BMI1 levels were normalized versus histone H3. (D) Interaction of pERK and BMI1; U2-OS/BMI2Py cells were stimulated with mitogen (stim) prior to IP; t+: longer exposure (IB: immunoblot; IP: immunoprecipitation). Parallel experiments with pP38 were inconclusive due to IB detection issues with applied antisera. [file 1756-8935-5-12-S1.tiff]

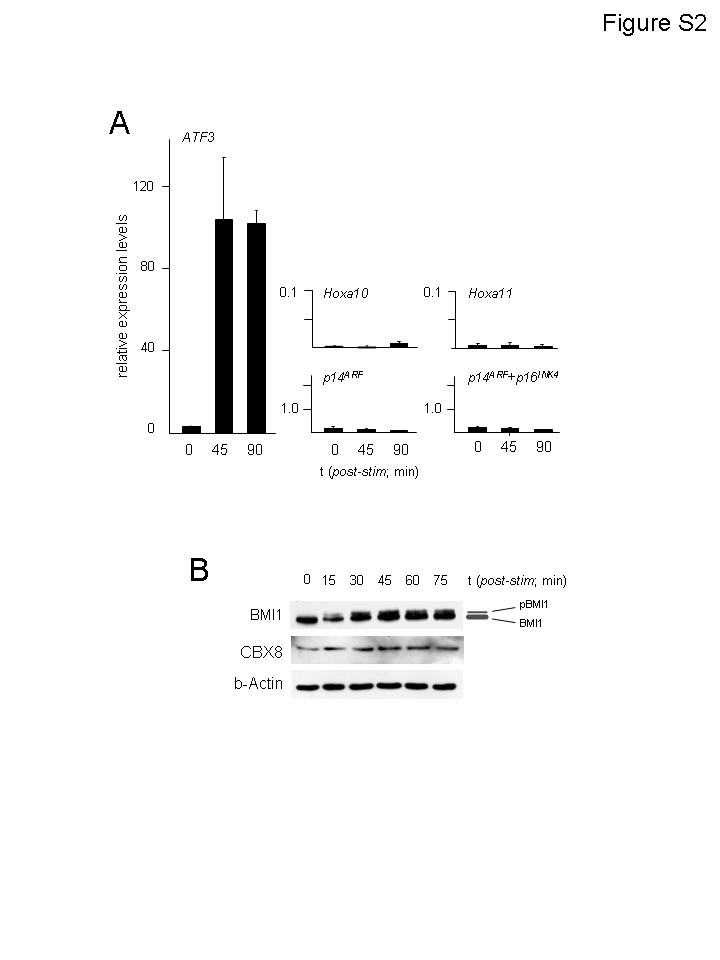

Supplement: Additional file 2 — Figure S2. PRC1/chromatin dissociation, not loss of H3K27me3, correlates with transcription. (A) mRNA expression of PRC1 target and non-target genes in TIG3 cells upon 0, 45 and 90 min of mitogen-stimulation. (B) Mitogenic stimulation induces BMI phosphorylation (upper panel; pBMI1) in TIG3 cells; cellular BMI1 and CBX8 protein (lower panel) levels remain unchanged under these conditions in support of changed PRC1/chromatin association rather than loss of protein (for example, degradation). [file 1756-8935-5-12-S2.tiff]

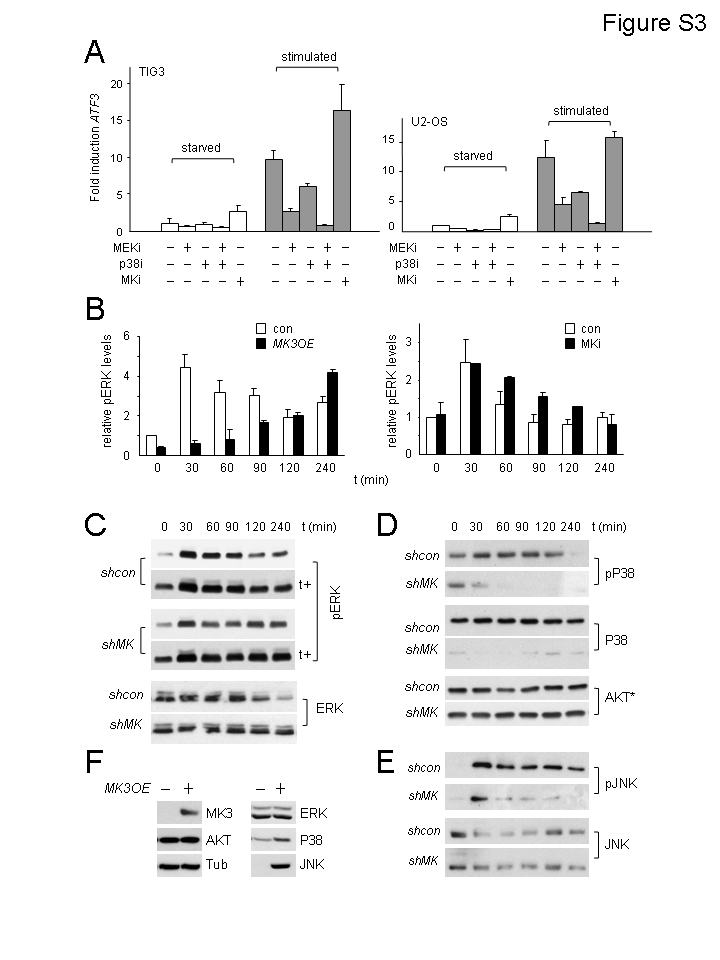

Supplement: Additional file 3 — Figure S3. MK3 is a negative regulator of ERK. (A) mRNA expression of PRC1 target gene ATF3 as a function of kinase inhibition in TIG3 cells (data as in Figure 3A) and U2-OS cells. (B) Quantification of pERK profiles (compare Figure 3B) in control versus MK3OE cells (left panel) and in control versus MKi cells (right panel); pERK levels were normalized to beta Actin (b-Actin). (C-F) IB-analysis of (p)ERK (C), (p)P38 (D), (p)JNK (E) in resting or mitogen-stimulated (stim) control and MK-knockdown (shMK) cells and (F) in the context of MK3OE; t (min): time post-stimulation in minutes. Samples corresponding to control (con) and experiment (MK3OE or shMK) were always loaded on the same gel to enable direct quantitative comparison; *: loading controls for all corresponding panels; t+: longer exposure. [file 1756-8935-5-12-S3.tiff]

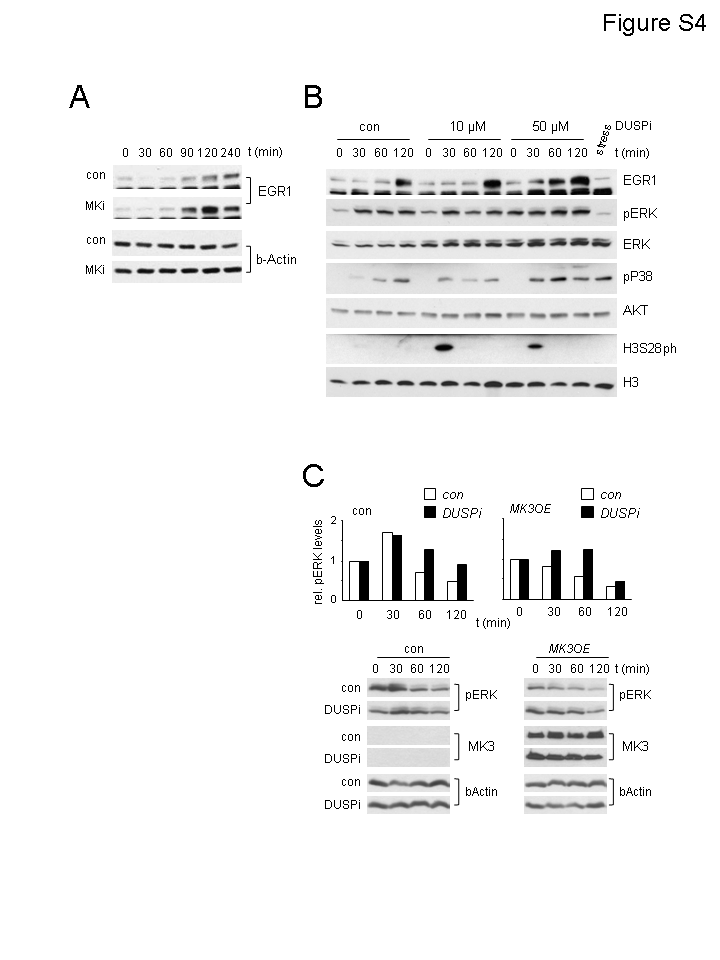

Supplement: Additional file 4 — Figure S4. DUSP involvement in regulation of MK3/ERK. (A) IB analysis of ERG1 in resting or mitogen-stimulated (stim) control (con) and MK-inhibited (MKi) cells; t (min): time post-stimulation in minutes. Samples corresponding to control and experiment (shMK) were always loaded on the same gel to enable direct quantitative comparison. (B) Cells were pretreated with DUSP inhibitor (DUSPi; concentration indicated) prior to mitogenic stimulation. IB detection of proteins as indicated. (C) Cells were pretreated with 50 μM DUSPi prior to mitogen stimulation. Samples corresponding to control and experiment (MK3OE) were always loaded on the same gel (bottom panel) to enable direct quantitative comparison. Quantitative analysis of effects of DUSPi in control cells (con; left panel) and MK3OE cells (right panel); normalization pERK levels was done versus beta Actin (b-Actin). [file 1756-8935-5-12-S4.tiff]

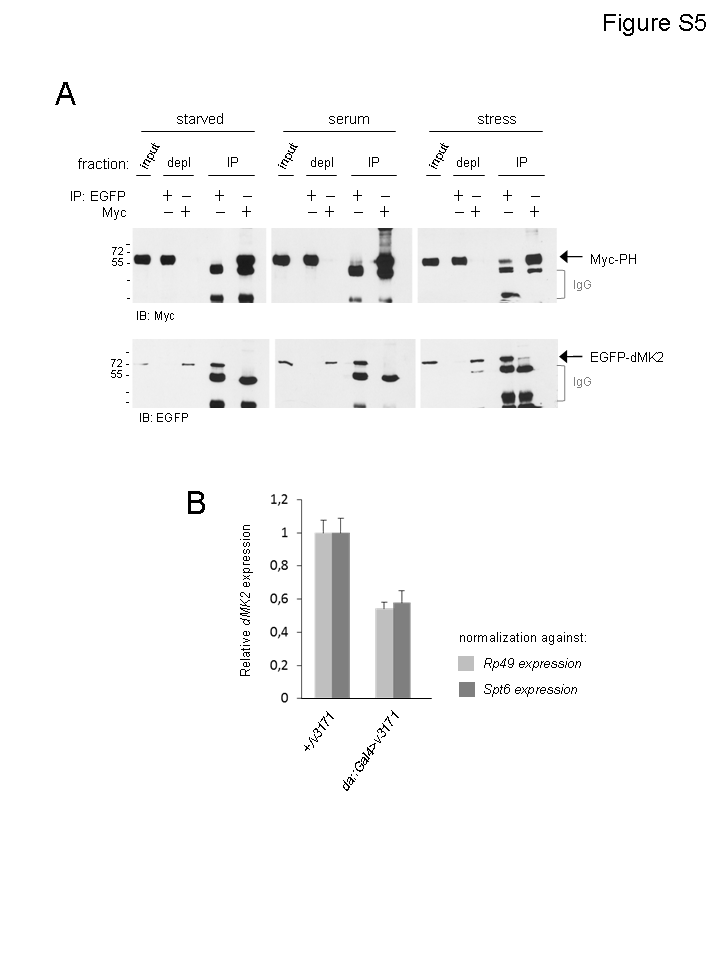

Supplement: Additional file 5 — Figure S5.PRC1/MK/ERK module represents a molecular switch mechanism. (A) Functional interaction between dMK2 and Polyhomeotic in vitro: stress signaling-induced interaction of EGFP-tagged dMK2 (Drosophila ortholog of MK2/3) and Myc-tagged Ph (Drosophila ortholog of PHC1/2) in S2 cells. The Myc-Ph sequences comprised the C-terminal SAM domain [54]. Conditions tested: starved, mitogen-stimulated (FCS/TPA) and stressed (arsenite). IgG represents IgG-heavy and -light chains. (B) Reduced dMK2 expression in third instar v3171 larvae; normalization indicated. [file 1756-8935-5-12-S5.tiff]
