# Supplementary material for: MK3 controls Polycomb target gene expression via negative feedback on ERK
Source: Epigenetics Chromatin. 2012 Aug 7;5:12. doi: 10.1186/1756-8935-5-12 (PMC3499388; doi:10.1186/1756-8935-5-12)
Supplement: Additional file 6 — Table S1.Antibodies used for ChIP, ICC, IB and IP. [file 1756-8935-5-12-S6.doc]

***Supplemental Table S2: antibodies used for ChIP, ICC, IB and IP***

| ChIP antisera | Species | µl/ChIP (~4µg) | Source |
| --- | --- | --- | --- |
| CBX8 | rabbit | 1 | Equal mix of α-CBX8 “LAST” and “GALD”; courtesy Klaus Hansen, Copenhagen, DK |
| H3K27me3 | rabbit | 4 | 07-449; Upstate Biotechnology/Millipore, Waltham, MA, USA |
| H3S28ph | rat (mono) | 40 | HTA28; courtesy Masaki Inagaki, Aichi, JP |
| HA | rabbit | 20 | sc-805; Santa Cruz Biotechnology, Santa Cruz, CA, USA |
| Rae28/PHC1 | rabbit | 4 | Rae28; Yoshihiro Takihara, Hiroshima, JP |
| ICC antisera | Species | Dilution | Source |
| BMI1 | mouse | 1:250 | F6; courtesy Maarten van Lohiuzen, Amsterdam, NL |
| H3S28ph | rat (mono) | 1:20 | See: ChIP antisera |
| IB antisera | Species | Dilution | Source |
| 2PY-tag | mouse | 1:300 | MMS-155R; Babco/Covance, Princeton, NJ,USA |
| BMI1 | mouse | 1:250-1000 | See: ICC sera |
| CBX4 | mouse | 1:25 | Clone M9; courtesy Arie Otte, Amsterdam, NL |
| CBX8 | rabbit | 1:5000 | See: ChIP antisera |
| MK3 | mouse | 1:500 | 3p8-1; gift from Stephan Ludwig, Münster, GE |
| b-Actin | mouse | 1:200.000 | C4, 69100, MP Biomedicals, Solon, OH, USA |
| AKT | rabbit | 1:1000 | 9272; Cell Signaling, Danvers, MA, USA |
| pERK (Thr202/Tyr204) | rabbit | 1:1000 | 9101; Cell Signaling, Danvers, MA, USA |
| ERK1/2 | rabbit | 1:1000 | 9102; Cell Signaling, Danvers, MA, USA |
| pP38 (Thr180/Tyr182) | rabbit | 1:1000 | 9211; Cell Signaling, Danvers, MA, USA |
| P38 | rabbit | 1:1000 | 9212; Cell Signaling, Danvers, MA, USA |
| pJNK (Thr183/Tyr185) | rabbit | 1:1000 | 9251; Cell Signaling, Danvers, MA, USA |
| JNK | rabbit | 1:1000 | 9252; Cell Signalling, Danvers, MA, USA |
| pMEK1 (T292) | rabbit (mono) | 1:1000 | GTX61817; Gene Tex, Irvine, CA, USA |
| pMEK1 (S298) | mouse | 1:100.000 | 558375; BD Pharmingen™/BD Biosciences, Franklin Lakes, NJ, USA |
| MEK1,2 | rabbit | 1:2000 | ABIN192427; Antibodies-online, Aachen, GE |
| H3 | rabbit | 1:5000 | ab1791; Abcam, Cambridge, UK |
| EGFP | mouse | 1:5000 | Roche Diagnostics, Basal, CH |
| MYC | mouse | 1:2500 | 9E10; Santa Cruz Biotechnology, Santa Cruz, CA, USA |
| H3S28ph H3  EGFP MYC | rat (mono) rabbit mouse mouse | 1:500 1:5000  ?? ?? | See: ChIP antisera |
| Tubulin | mouse | 1:10.000 | T6074, Sigma-Aldrich, St. Louis, MO, USA |
| IP antisera | Species | µl/IP | Source |
| 2Py-tag | mouse | 3 | See: IB antisera |
| EGFP | mouse | 3 µg | See: IB antisera |
| MYC | mouse | 3 µg | See: IB antisera |
