# Supplementary material for: MK3 controls Polycomb target gene expression via negative feedback on ERK
Source: Epigenetics Chromatin. 2012 Aug 7;5:12. doi: 10.1186/1756-8935-5-12 (PMC3499388; doi:10.1186/1756-8935-5-12)
Supplement: Additional file 7 — Table S2. Primer sequences and targeting sequences. [file 1756-8935-5-12-S7.doc]

***Supplemental Table S1: primer sequences and targeting sequences***

| Cloning primers –Taqman | Fw/Rev | sequence (5 ’→ 3’) |
| --- | --- | --- |
| *dMK2* | Forward | CACCatgctttctctgcagaatcaacg TGCGCGTCGCATTGGCCATGTATAACTC. |
|  | Reverse | GTTGCGCGTCGCATTGGCCATGTATAACTC |
| *PH* | Forward | caccatgacaacgatcaccaatgg |
|  | Reverse | CTGCGCTCCTGGATGCTTGGCCTC |
| shRNA target sequences | Number | sequence (5 ’→ 3’) |
| *MK3* | #3 | GGAGGAGATGACCAGTGCC |
| PCR primers for ChIP analysis | Fw/Rev | sequence (5 ’→ 3’) |
| *p14ARF exon1* | Forward | GTGGGTCCCAGTCTGCAGTTA |
|  | Reverse | CCTTTGGCACCAGAGGTGAG |
| *15kb downstream of ARFpromoter* | Forward | GCACTTGCCCTTCCAGGTATA |
|  | Reverse | TGATAGTTCAAGGCCCTATGCC |
| *p16INK4A promoter* | Forward | ACCCCGATTCAATTTGGCAG |
|  | Reverse | AAAAAGAAATCCGCCCCCG |
| *p16INK4A exon1* | Forward | AGAGGGTCTGCAGCGG |
|  | Reverse | TCGAAGCGCTACCTGATTCC |
| *CCNA2* | Forward | TGACGTCATTCAAGGCGACAG |
|  | Reverse | GCTCAGTTTCCTTTGGTTTACCC |
| *p15 exon1* | Forward | GGAACCTAGATCGCCGATGTAG |
|  | Reverse | TGTTTTACGCGTGGAATGCAC |
| *ATF3* | Forward | TGTTTTTTCTTTTGCGTTTGGC |
|  | Reverse | TCGTGGCAACCAAATCTAAACAG |
| *HOXA10* | Forward | CCCGAGCTGATGAGCGAGTC |
|  | Reverse | GCCAAATTATCCCACAACAATGTC |
| *HOXA11* | Forward | AATCTATCCCCATCCTTAGCAGG |
|  | Reverse | TTGTCAATTTCAACATCGGGTC |
| primer sequences for rtPCR analysis | Fw/Rev | sequence (5 ’→ 3’) |
| *p14ARF* | Forward | CCCTCGTGCTGCTGATGCTACTG |
|  | Reverse | CCCATCATCATGACCTGGTCTT |
| *p14ARF+p16INK4A* | Forward | GAAGGTCCCTCAGACATCCCC |
|  | Reverse | CCCTGTAGGACCTTCGGTGAC |
| *cyclophillin A* | Forward | TTCCTGCTTTCACAGAATTATTCC |
|  | Reverse | GCCACCAGTGCCATTATGG |
| *ATF3* | Forward | TCACTGTCAGCGACAGACCC |
|  | Reverse | CTACCTCGGCTTTTGTGATGG |
| *HOXA10* | Forward | GCCCTTCCGAGAGCAGCAAAG |
|  | Reverse | AGGTGGACGCTGCGGCTAATCTCTA |
| *HOXA11* | Forward | TGCCAAGTTGTACTTACTACGTC |
|  | Reverse | GTTGGAGGAGTAGGAGTATGTCA |
| *CCNA2* | Forward | CGCTCCAAGAGGACCAGGA |
|  | Reverse | CCCTCCTGCAGATATCCCG |
| *b-Actin* | Forward | CCTGGCACCCAGCACAAT |
|  | Reverse | GCCGATCCACACGGAGTACT |
| *dMK2* | Forward | CCTGAGGTTCTGGGCCCGGA |
|  | Reverse | AGAACGGCGGGAATCCGCAC |
| *rp49* | Forward | CCGCTTCAAGGGACAGTATC |
|  | Reverse | GACAATCTCCTTGCGCTTCT |
| *spt6* | Forward | CGGAGGAGCTCTTCGATATG |
|  | Reverse | GACAGCTCTGGGAAGTCGTC |
